# Transcatheter Aortic Valve Implantation with Embolic Protection System in a Patient with Left Ventricle Apical Thrombus

**DOI:** 10.5935/abc.20170109

**Published:** 2017-11

**Authors:** João Gonçalves Almeida, Sara Ferreira, Daniel Caeiro, José Ribeiro, Vasco Gama Ribeiro

**Affiliations:** 1 Centro Hospitalar Vila Nova de Gaia/Espinho, Vila Nova de Gaia, Portugal; 2 Hospital Divino Espirito Santo, Ponta Delgada, Portugal

**Keywords:** Heart Valve Prosthesis Implantation, Embolic Protection Devices, Shock, Cardiogenic

**Figure 1:**
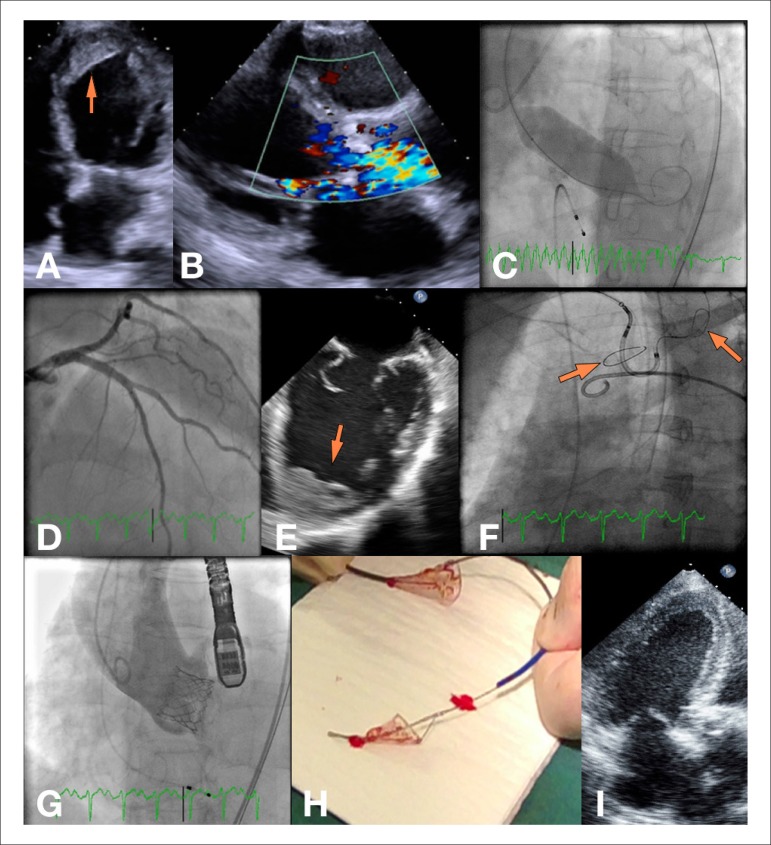
A) Four-chamber view from the admission TTE (arrow: apical thrombus); B) Colour Doppler showing turbulent flow through the aortic valve in parasternal long-axis view; C) Percutaneous aortic balloon valvuloplasty; D) Left coronary angiography; E) TEE showing the large apical thrombus (arrow); F) Embolic protection system deployment (arrows: filters); G) Angiography after TAV implantation; H) Embolic filters with particulate debris; I) Three-chamber view from a TTE, 3-months after the procedure. TTE: transthoracic echocardiography; TEE: transoesophageal echocardiography; TAV: transcatheter aortic valve.

A 68-year-old woman was admitted in our acute cardiac care unit due to cardiogenic shock. The transthoracic echocardiography (TTE) showed severe aortic stenosis, severe left ventricle (LV) systolic dysfunction (ejection fraction 20%) and a large apical thrombus (Figure 1A-B). We performed an emergent percutaneous aortic balloon valvuloplasty (Figure 1C). During the procedure, the coronary angiography revealed no epicardial coronary disease (Figure 1D). Despite some mild clinical and hemodynamic improvement (mean gradient reduced from 40 to 30 mmHg), she remained in New York Heart Association (NYHA) class IV.

The case was discussed by our heart team and she was considered to be at high operative risk (Society of Thoracic Surgery score 12%; EUROSCORE II 15%). Therefore, we have decided to implant a transcatheter aortic valve (TAVI) using an embolic protection system. Aortic annulus sizing was performed intra-procedure using transoesophageal echocardiography, which also showed the apical thrombus (Figure 1E). Firstly, the Sentinel Cerebral Protection System (Claret Medical, Inc) was deployed through right radial access (Figure 1F). Afterwards, a 26 mm Edwards Sapien 3 TAV (Edwards Lifesciences Corporation) was implanted by transfemoral approach (Figure 1G). The procedure went without complications and the patient showed remarkable clinical and hemodynamic improvement, being discharged 11 days after TAVI, medicated with warfarin. In the one-year follow-up, the patient was in NYHA class I, TTE showed normally functioning TAV, improvement of the LV function (40%) and no evidence of apical thrombus (Figure 1I).

